# Developmental programming of somatic growth, behavior and endocannabinoid metabolism by variation of early postnatal nutrition in a cross-fostering mouse model

**DOI:** 10.1371/journal.pone.0182754

**Published:** 2017-08-31

**Authors:** Felix Schreiner, Merle Ackermann, Michael Michalik, Eva Hucklenbruch-Rother, Andras Bilkei-Gorzo, Ildiko Racz, Laura Bindila, Beat Lutz, Jörg Dötsch, Andreas Zimmer, Joachim Woelfle

**Affiliations:** 1 Pediatric Endocrinology Division, Children’s Hospital, University of Bonn, Bonn, Germany; 2 Department of Pediatric and Adolescent Medicine, Medical Faculty, University of Cologne, Cologne, Germany; 3 Department of Molecular Psychiatry, University Hospital Bonn, Bonn, Germany; 4 Institute for Physiological Chemistry, University Medical Center of the Johannes Gutenberg University, Mainz, Germany; Faculty of Medicine & Health Science, UNITED ARAB EMIRATES

## Abstract

**Background:**

Nutrient deprivation during early development has been associated with the predisposition to metabolic disorders in adulthood. Considering its interaction with metabolism, appetite and behavior, the endocannabinoid (eCB) system represents a promising target of developmental programming.

**Methods:**

By cross-fostering and variation of litter size, early postnatal nutrition of CB6F1-hybrid mice was controlled during the lactation period (3, 6, or 10 pups/mother). After weaning and redistribution at P21, all pups received standard chow ad libitum. Gene expression analyses (liver, visceral fat, hypothalamus) were performed at P50, eCB concentrations were determined in liver and visceral fat. Locomotor activity and social behavior were analyzed by means of computer-assisted videotracking.

**Results:**

Body growth was permanently altered, with differences for length, weight, body mass index and fat mass persisting beyond P100 (all 3>6>10,p<0.01). This was paralleled by differences in hepatic IGF-I expression (p<0.01). Distinct gene expression patterns for key enzymes of the eCB system were observed in fat (eCB-synthesis: 3>6>10 (DAGLα p<0.05; NAPE-PLD p = 0.05)) and liver (eCB-degradation: 3>6>10 (FAAH p<0.05; MGL p<0.01)). Concentrations of endocannabinoids AEA and 2-AG in liver and visceral fat were largely comparable, except for a borderline significance for higher AEA (liver, p = 0.049) in formerly overfed mice and, *vice versa*, tendencies (p<0.1) towards lower AEA (fat) and 2-AG (liver) in formerly underfed animals. In the arcuate nucleus, formerly underfed mice tended to express more eCB-receptor transcripts (CB1R p<0.05; CB2R p = 0.08) than their overfed fellows. Open-field social behavior testing revealed significant group differences, with formerly underfed mice turning out to be the most sociable animals (p<0.01). Locomotor activity did not differ.

**Conclusion:**

Our data indicate a developmental plasticity of somatic growth, behavior and parameters of the eCB system, with long-lasting impact of early postnatal nutrition. Developmental programming of the eCB system in metabolically active tissues, as shown here for liver and fat, may play a role in the formation of the adult cardiometabolic risk profile following perinatal malnutrition in humans.

## Introduction

Referring to the concept of impaired developmental programming as initially proposed by David Barker and co-workers [1], low birth weight and growth restriction during critical periods in development have been associated with the predisposition to metabolic disorders in numerous epidemiological studies [2],[3]. Importantly, excessive early postnatal weight gain thereafter, or rather rapid catch-up growth, has also been associated with an adverse metabolic phenotype later in life, highlighting the close interaction between growth and metabolism [4, 5].

One of the most intensively studied endocrine cascades assumed to be involved in developmental programming of growth and metabolism is the GH-IGF-I-system. At birth, children with intrauterine growth retardation frequently show decreased hepatic protein synthesis including IGF-I [6]. Postnatally, after nutrient deprivation has been resolved, IGF-I-levels increase and correlate well with the individual catch-up growth, while persistent abnormalities of GH and/or IGF-I secretion have been observed in those individuals with lacking or only incomplete catch-up growth [7, 8]. Furthermore, in monozygotic twins with discordant birth weight due to twin-to-twin-transfusion syndrome (TTTS), IGF-I levels at birth have been shown to be predictive of later catch-up growth capacity [9]. Recent animal studies support the assumption of a developmental plasticity of the GH/IGF-I–axis following modification of early postnatal nutrient intake, showing long-term effects not only on somatic growth, but also on cardiometabolic parameters [10].

Considering its close relationship with energy homeostasis and food intake, the endocannabinoid (eCB) system represents a promising target of early developmental programming. Endocannabinoid action is mediated by two different receptors, CB1R and CB2R, and their endogenous ligands, among which anandamide and 2-arachidonoyl glycerol (2-AG) are the most important molecules. Whereas CB2R is mainly expressed in immune cells, CB1R is broadly expressed at central and peripheral levels and is deeply implicated in the regulation of several neuropsychological, endocrine, and metabolic functions [11]. Activation of central CB1R stimulates appetite and food intake [12, 13]. Furthermore, CB1R knockout mice are not only leaner compared to wildtype animals when fed a standard diet, they also show a relative resistance to diet-induced obesity and obesity-related insulin and leptin resistance even though total caloric intake is hardly different from that of wildtype animals fed the same diet [14–16]. Accordingly, clinical trials using the inverse CB1R-agonist rimonabant (SR141716A) yielded promising results in terms of weight loss and improvement of the metabolic profile in obese patients [17]. However, undesirable psychiatric side effects led to its removal, while several laboratories now put in great efforts to develop selective peripherally acting agents [18]. There is much to indicate that a dysregulation of the eCB system, which is found to be generally upregulated in obesity, may be causally implicated in the pathogenesis of the metabolic syndrome [19]. However, although certain nutrient interventions with ω-3-polyunsaturated fatty acids that lower the availability of endocannabinoid precursors have been shown effective in reversing some aspects of eCB dysregulation, the mechanisms originally leading to the imbalance of the eCB system are largely unknown [20]. Environmental factors such as stress and nutrition during early life are increasingly considered as triggers of disturbed physiological and behavioral functions in adulthood [21] and could also have long-term impact on the eCB system.

Here, we hypothesized that variation of early postnatal nutrition is not only able to permanently modify body growth, but also affects eCB homeostasis in metabolically active tissues. Postnatal nutrition was modified by variation of the litter size within naturally occurring ranges (3–10 pups per mother during lactation). This led to a permanent imprint of somatic growth, social behavior, and parameters of the endocannabinoid metabolism in liver, adipose tissue, and the hypothalamic arcuate nucleus.

## Material and methods

### Cross-fostering mouse model and animal care

Pregnant mice from C57BL/6 (father) and Balb/c (mother) matings were purchased from Charles River Laboratories (Sulzberg, Germany) and arrived at embryonic age 16 days (E16). First generation CB6F1 hybrid mice are genetically uniform like inbred strains and known to be physiologically robust. Four days after arrival (E20), mothers delivered on average 6.7 ± 1.7 CB6F1 hybrid newborns. 24 to 36 hours after birth, newborns were cross-fostered—that is, they were systematically redistributed into groups of 3, 6 or 10 newborns per mother. To avoid biased maternal care that may result from co-housing of biological and fostered pups, none of the newborn animals remained together with its biological mother. Survival rate until postnatal day 6 (P6) was 91.2%, with the majority of deceased pups belonging to the undernutrition group. Thereof, we used only those animals from nutrition groups with at least 9 surviving pups per mother (referred to as 10/mother group) for further experiments, whereas one group with only 8 surviving animals was excluded, since auxological development in this group appeared to be more similar to the 6/mother control group. Between P7 and P21 survival rate was thoroughly 100% in all nutrition groups. At P21, mice were separated from their mothers and redistributed again. Thereafter, females were housed in groups of 4 animals per cage (at least one mouse from each nutrition group per cage) whereas males were kept solitarily in order to avoid rivalry and aggression, which may complicate cohousing of males who did not grow up together.

All animals were housed under pathogen-free conditions in individually ventilated filter cages with a 12/12-hr dark/light cycle with constant temperature (22°C) and free access to standard chow and water. At P50, mice were sacrificed (CO_2_ exposure followed by cervical dislocation) after a 4–6 h fasting period. Organs were weighed and immediately frozen for storage at -80°C. In addition to the main animal cohort, which was sacrificed at P50, a second set of animals was used for non-interventional experiments (behavior testing) and further follow-up of auxological development until P100. Cross-fostering conditions and main auxological outcome parameters were identical between the two animal sets (not shown).

Body length was determined by measuring the naso-anal distance. Body mass index (BMI) was calculated as the ratio between body weight and square body surface area [g/m^2^]. Body surface area was estimated by using the *DuBois*-formula: body surface [m^2^] = 0.007184 x weight [kg^0.425^] x height [cm^0.725^].

### RNA isolation and real-time PCR

For expression analyses, we used only male animals because of a known impact of the menstrual cycle on several parameters of the eCB system, including circulating endocannabinoids and the hypothalamic expression of CB1R [22, 23].

RNA from liver tissue was isolated using the TRIzol reagent, according to the manufacturer’s recommendations (Life Technologies, Karlsruhe). Because of the high lipid content, RNA extraction from white adipose tissue was performed by a commercially available kit protocol (RNeasy Mini Kit, Qiagen, Hilden, Germany) after disruption using mortar and pestle with liquid nitrogen and subsequent homogenization in a microcentrifuge spin column shredding system (QiaShredder, Qiagen, Hilden, Germany). RNA from hypothalamic arcuate nuclei was isolated using the RNAeasy Mini Kit, according to the manufacturer’s protocol. Because of the relatively low tissue amount, we pooled left and right nucleus of each animal.

1 μg (arcuate nucleus: 0.3 μg) isolated RNA was reverse transcribed into cDNA by a commercially available reverse transcriptase (Superscript VILO cDNA Synthesis Kit, Invitrogen, Karlsruhe, Germany). In order to avoid inaccuracies due to pipetting volumes below 2.0 μl, obtained cDNA solutions were diluted (1:1 to 1:2) prior to real-time PCR reactions (standard cDNA pipetting volume was 3.0 μl per PCR reaction). Real-time PCR was performed on a Roche Light Cycler 480 platform (Roche, Penzberg, Germany) using SYBR Green fluorescence dye (SYBR Green I Master Mix, Roche). Primer sequences are given in the *supplementary material* (S1 Table). All reactions were run in duplicates. PCR reaction mix and cycling conditions were adjusted to the manufacturer’s recommendation. Relative expression was calculated by the 2(-Delta Delta C(T)) -method [24].

### Endocannabinoid extraction and quantification

Extraction and LC/MRM-quantification (Liquid Chromatography/Multiple Reaction Monitoring) of the endocannabinoids anandamide (AEA) and 2-arachidonoyl glycerol (2 AG) as well as arachidonic acid (AA) from frozen (-80°C) liver and visceral (perigonadal) adipose tissue was performed as decribed elsewhere [25]. eCB concentrations were normalized to protein content for statistical analyses.

### Social behavior testing

Social interaction activity was analyzed at age P45-P50 in male animals (n = 6–8 per nutrition group) in an open-field setting [26, 27], using two cage-in-cage boxes (S3 Fig) placed at opposite corners of the open-field-apparatus (44x44x30 cm). One box contained a ‘partner animal’, the other was empty and served as control ‘object’. Movements of the animal were recorded by a computer-assisted videotracking system (TSE Systems, Bad Homburg, Germany). Locomotor activity was measured by calculating duration and distance that the animal travelled during the test period of 10 min. Behavioral tests were performed during morning to forenoon (approx. 3–5 h after start of the light phase, with thorough mixing of animal order and nutrition group allocation, no explicit prior fasting period).

### Statistical analyses

Data are presented as mean ± SEM. Statistical analyses were performed using the SPSS software package, version 20 (SPSS Inc., Chicago, IL, USA). Differences between nutrition groups were analyzed by ANOVA, effect sizes were assessed by linear regression. For parameters which were not normally distributed we used the non-parametric Kruskal-Wallis- and Mann-Whitney-U-tests. Generally, statistical significance was assumed for P values <0.05.

### Ethics

All animal experiments were performed in accordance with the European guidelines for the care and use of laboratory animals. The study was approved by the animal care ethics committee of the University of Bonn and the responsible state ministry (LANUV).

## Results

### Somatic growth and BMI is permanently altered by early postnatal nutrition

**Fig 1:**
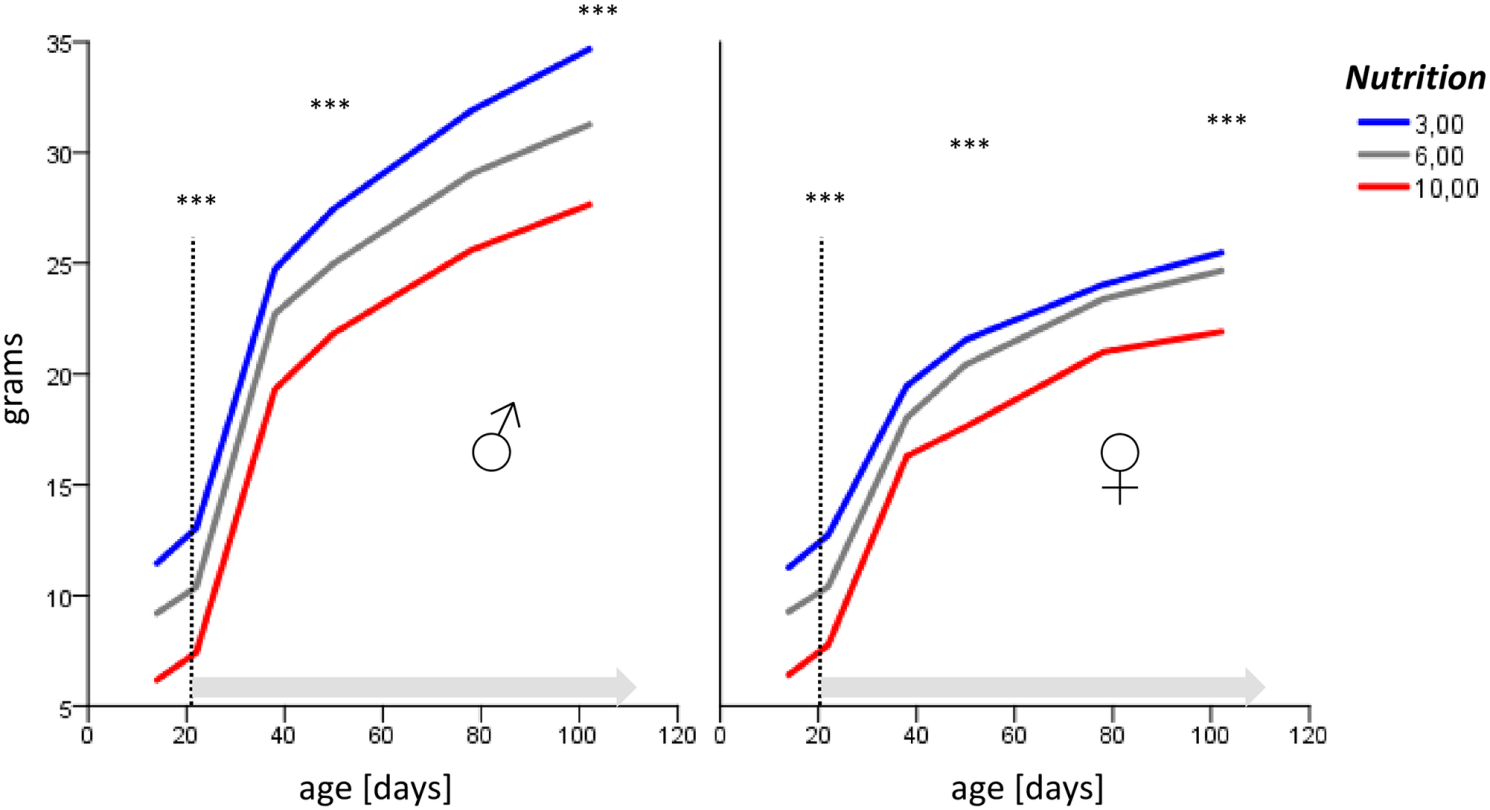
Body weight in grams (y-axis) and age in days of postnatal development (x-axis), longitudinal data of a P100 cohort. Note that differences between the nutrition groups (3, 6, and 10 pups per mother, respectively) persisted after weaning and redistribution at P21 (dotted line), after which mice had free access to standard food.

**Fig 2:**
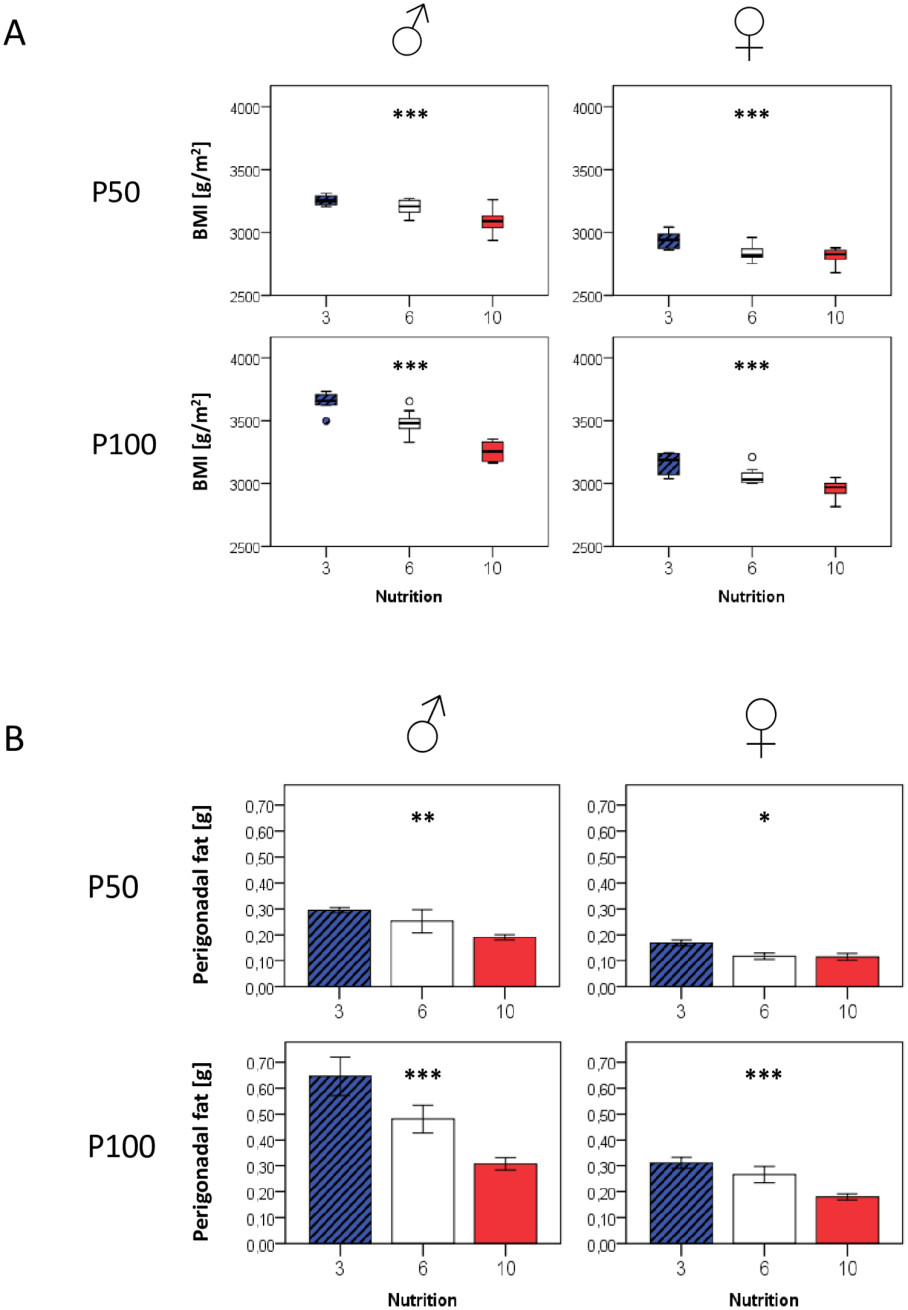
BMI (Fig. 2A) and perigonadal fat mass (Fig. 2B) at P50 and P100 in relation to postnatal nutrition and gender. Group differences for both parameters tended to further increase with age. If not indicated otherwise, displayed significance levels (* p<0.05, ** p<0.01, *** p<0.001) refer to comparisons between all three nutrition groups (ANOVA). P50: n = 4–10 animals per nutrition group and gender; P100: n = 5–10 animals per nutrition group and gender.

At the end of the cross-fostering period (P21), there were significant differences for body weight and length between the nutrition groups. Notably, differences persisted beyond age P100 (Fig 1 and S4 Fig), albeit growth curves in females differed slightly less than in males. Comparable group differences in both genders were also seen for BMI (Fig 2A) and perigonadal fat mass (Fig 2B, with a strong overall correlation between fat mass and total body weight (R 0.79, p < 0.001). Importantly, none of the mice can be defined as malnourished or obese until P100, so that we chose to designate them as formerly (relatively) underfed, control, and overfed animals, respectively.

**Fig 3:**
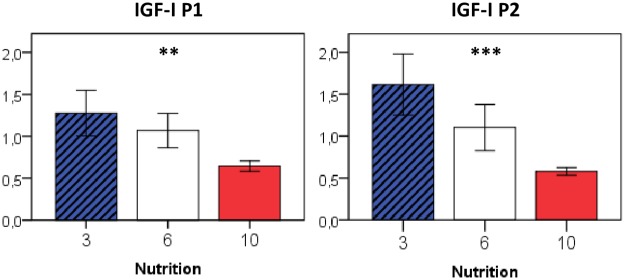
Relative gene expression of IGF-I in the liver at P50 in relation to early postnatal nutrition. If not indicated otherwise, displayed significance levels (** p<0.01, *** p<0.001) refer to comparisons between all three nutrition groups (ANOVA). n = 4–6 males per nutrition group.

Somatic growth patterns were paralleled by differences in hepatic IGF-I expression, as shown for mRNA transcripts derived from both known IGF-I promotors P1 and P2 (Fig 3).

### Effects on key enzymes of the eCB system in fat and liver tissue

In order to obtain a comprehensive picture of the eCB metabolism in adipose tissue and liver, we analyzed mRNA expression levels of eCB-synthesizing (NAPE-PLD, DAGLα) and eCB–degrading (FAAH, MGL) enzymes and the CB1R, in addition to LC/MS-measurements of endocannabinoid concentrations in liver and white adipose tissue.

**Fig 4:**
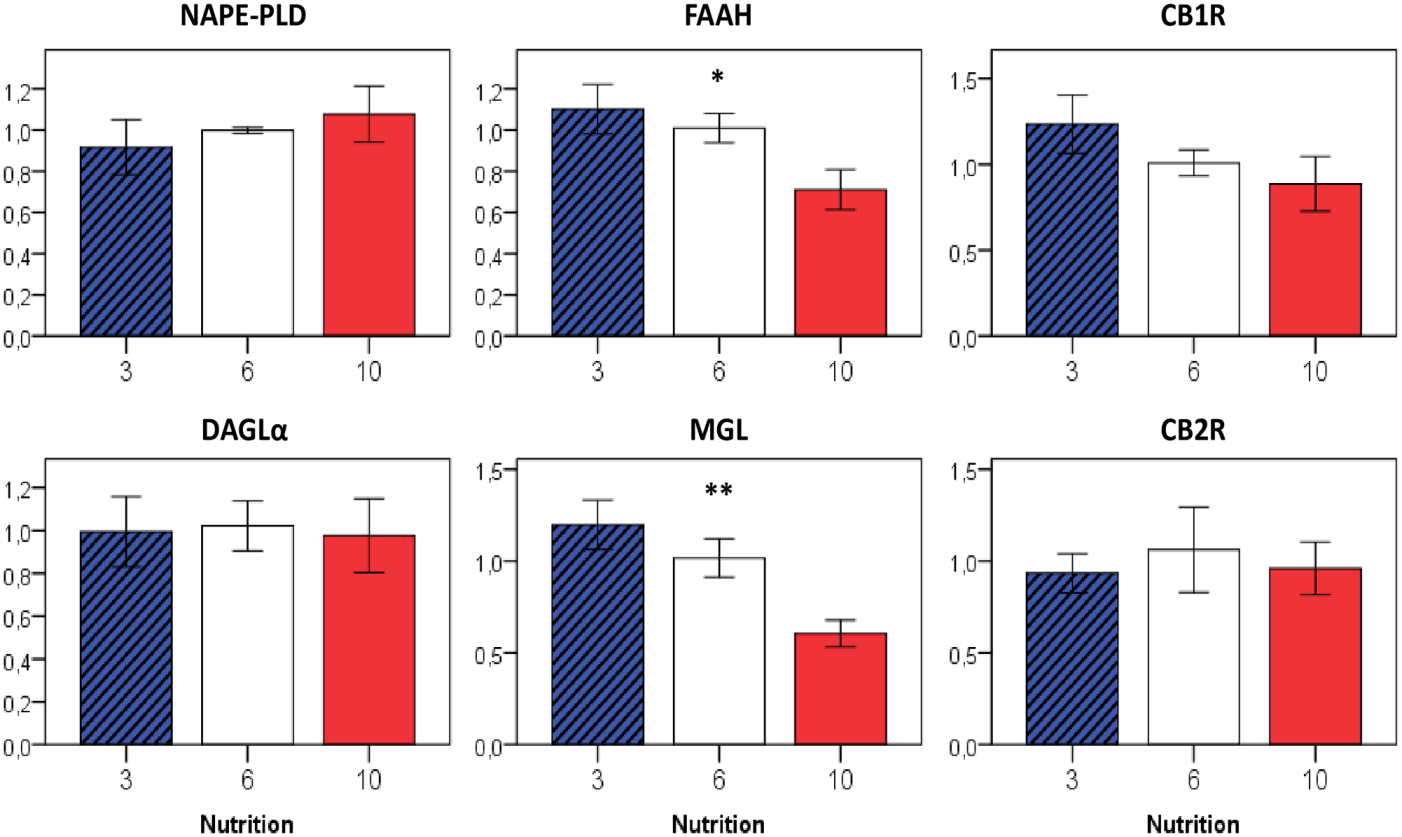
Relative gene expression of eCB-metabolizing enzymes in the liver at P50 in relation to early postnatal nutrition. If not indicated otherwise, displayed significance levels (* p<0.05, ** p<0.01) refer to comparisons between all three nutrition groups (ANOVA). n = 4–6 males per nutrition group.

**Fig 5:**
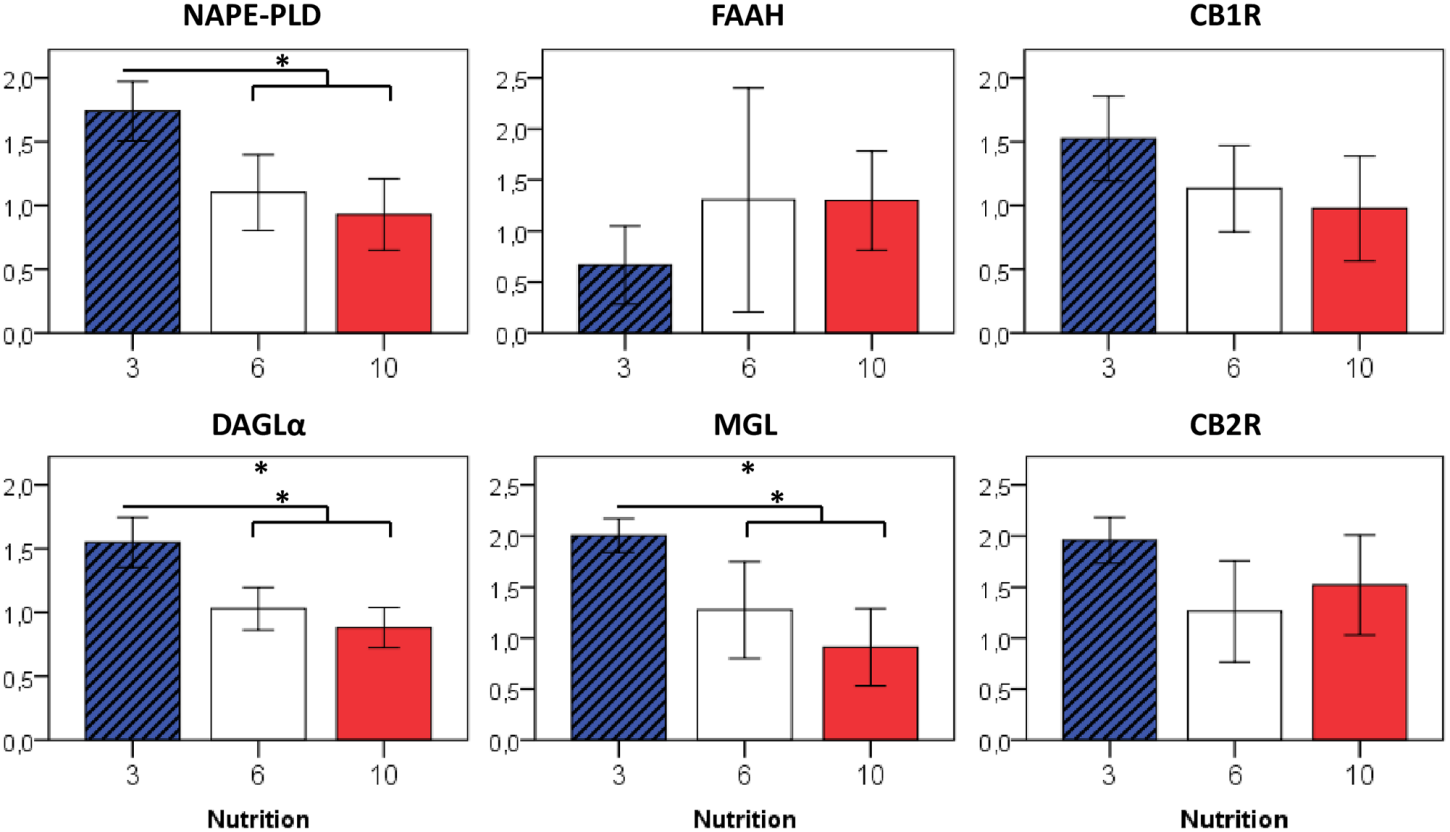
Relative gene expression of eCB-metabolizing enzymes in visceral (perigonadal) adipose tissue at P50 in relation to early postnatal nutrition. n = 4–6 males per nutrition group, * p<0.05 (ANOVA).

eCB-synthesizing enzymes in white adipose tissue tended to be higher expressed in formerly overfed mice (3>6>10: DAGLα p = 0.02, NAPE-PLD p = 0.06; 3 vs. 6/10: DAGLα p = 0.03, NAPE-PLD p = 0.02), whereas no differences were observed in the liver (Figs 4 and 5). Enzymes involved in the eCB-degradation showed a similar distribution, with highest expression levels seen in formerly overfed animals in both adipose tissue (3>6>10: MGL p = 0.03) and liver (3>6>10: FAAH p = 0.01 and MGL p < 0.01). For CB1R, no expression differences were observed, neither in adipose tissue nor in the liver (Figs 4 and 5).

**Fig 6:**
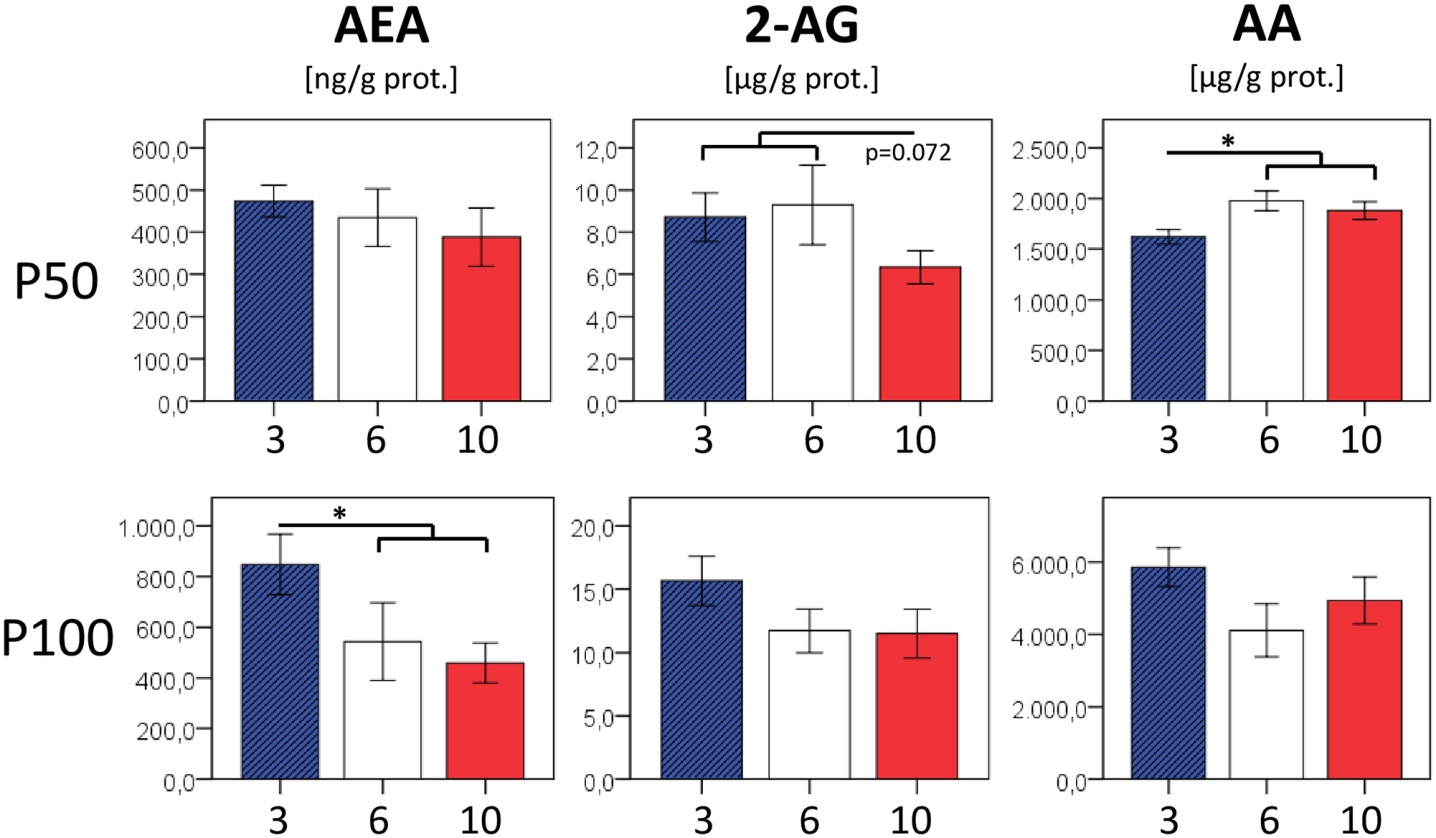
Concentrations of endocannabinoids anandamide (AEA) and 2-arachidonoyl glycerol (2 AG) as well as arachidonic acid (AA) in liver tissue. In addition to higher AEA (P100, p = 0.049) in formerly overfed animals and a trend towards lower 2-AG concentrations in formerly underfed animals (P50, p = 0.072), we observed a significantly reduced total amount of the eCB component arachidonic acid (AA) in the former overfed group (3 vs. 6/10 p = 0.023). n = 4–7 males per nutrition group.

**Fig 7:**
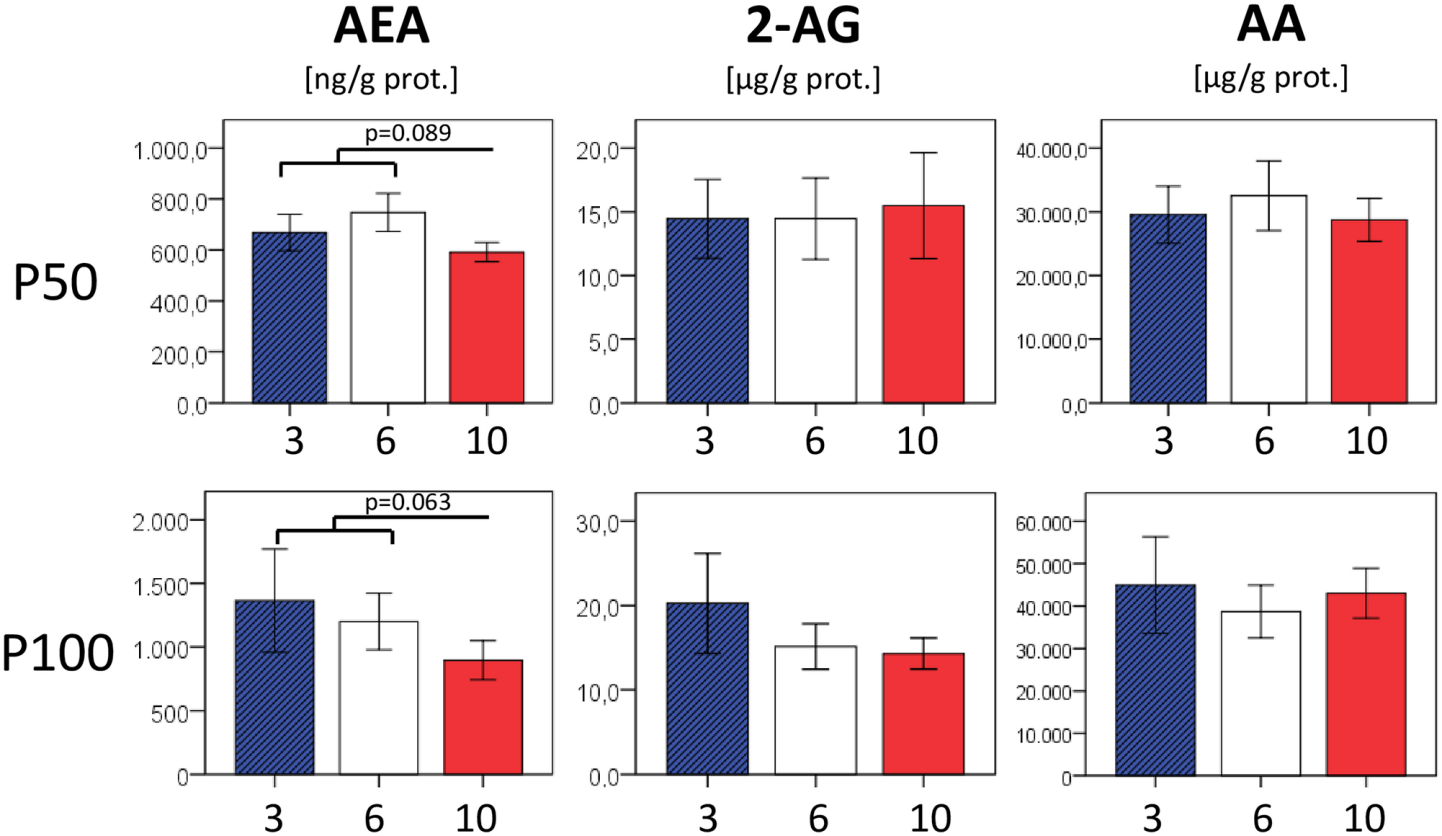
Concentrations of endocannabinoids anandamide (AEA) and 2-arachidonoyl glycerol (2 AG) as well as arachidonic acid (AA) in visceral (perigonadal) adipose tissue. Except for tendencies towards lower AEA concentrations in formerly underfed animals (P50: 10 vs. 3/6 p = 0.089; P100: 10 vs.3/6 p = 0.063) no differences between former nutrition groups were detected at P50 and P100, resp. n = 4–6 males per nutrition group.

At P50, eCB concentrations in the liver and in white adipose tissue did not show significant differences between the nutrition groups, except for trends towards lower 2-arachidonoyl glycerol (10 vs. 3/6, p = 0.072) in the liver and lower anandamide levels in fat samples (10 vs. 3/6, p = 0.089) of the formerly food restricted group, respectively. In an additional sample set taken at P100, we noted borderline significances for increased AEA (liver, p = 0.049) in formerly overfed mice and decreased AEA in formerly underfed mice (fat, p = 0.063) (Figs 6 and 7).

### Effect on hypothalamic eCB-receptor expression

**Fig 8:**
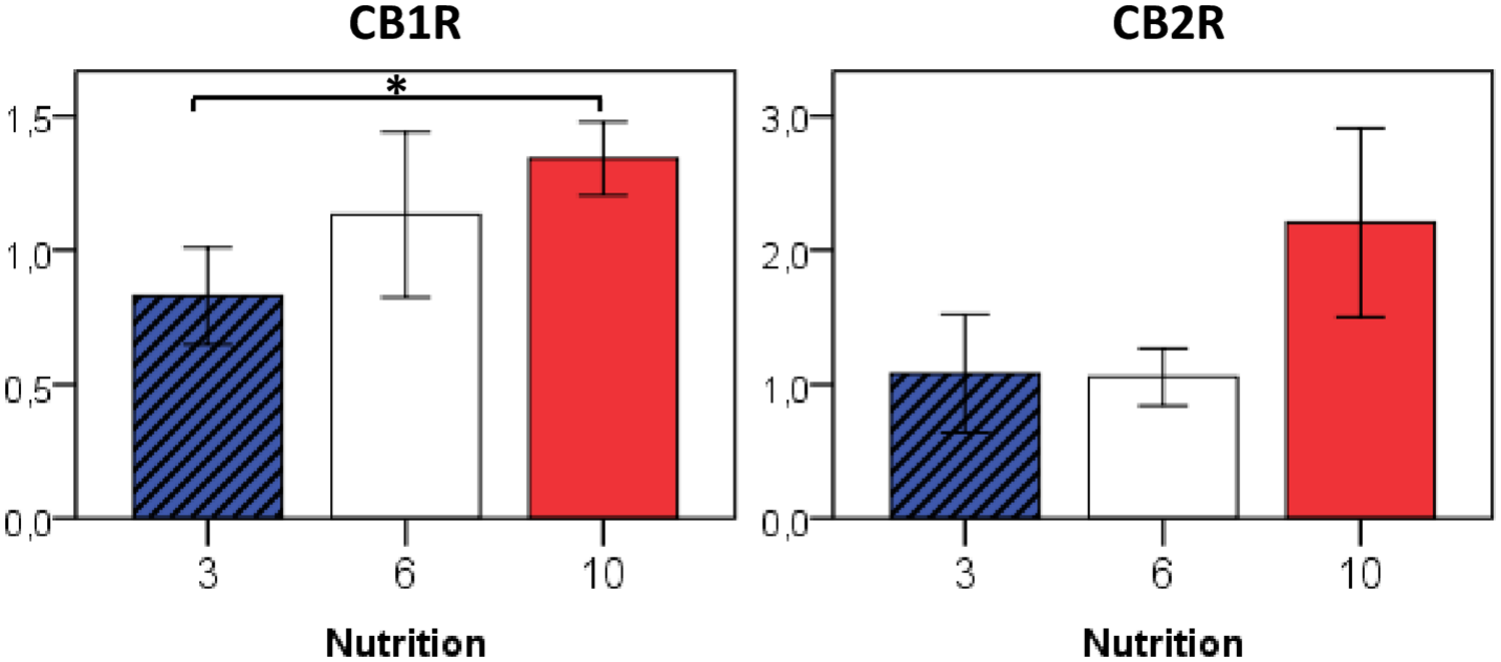
Relative gene expression of eCB receptors in the hypothalamic arcuate nucleus at P50 in relation to early postnatal nutrition. n = 4–5 males per nutrition group, * p<0.05 (ANOVA).

Compared to fat and liver tissue, we analyzed only a reduced number of genes in the hypothalamic arcuate nucleus because of the lower amount of starting tissue and available RNA. Formerly underfed mice tended to express more eCB-receptor transcripts (CB1R: 10 vs. 3: p = 0.04; CB2R 10 vs. 3/6: p = 0.08) than their overfed fellows (Fig 8).

### Effects on social behavior

**Fig 9:**
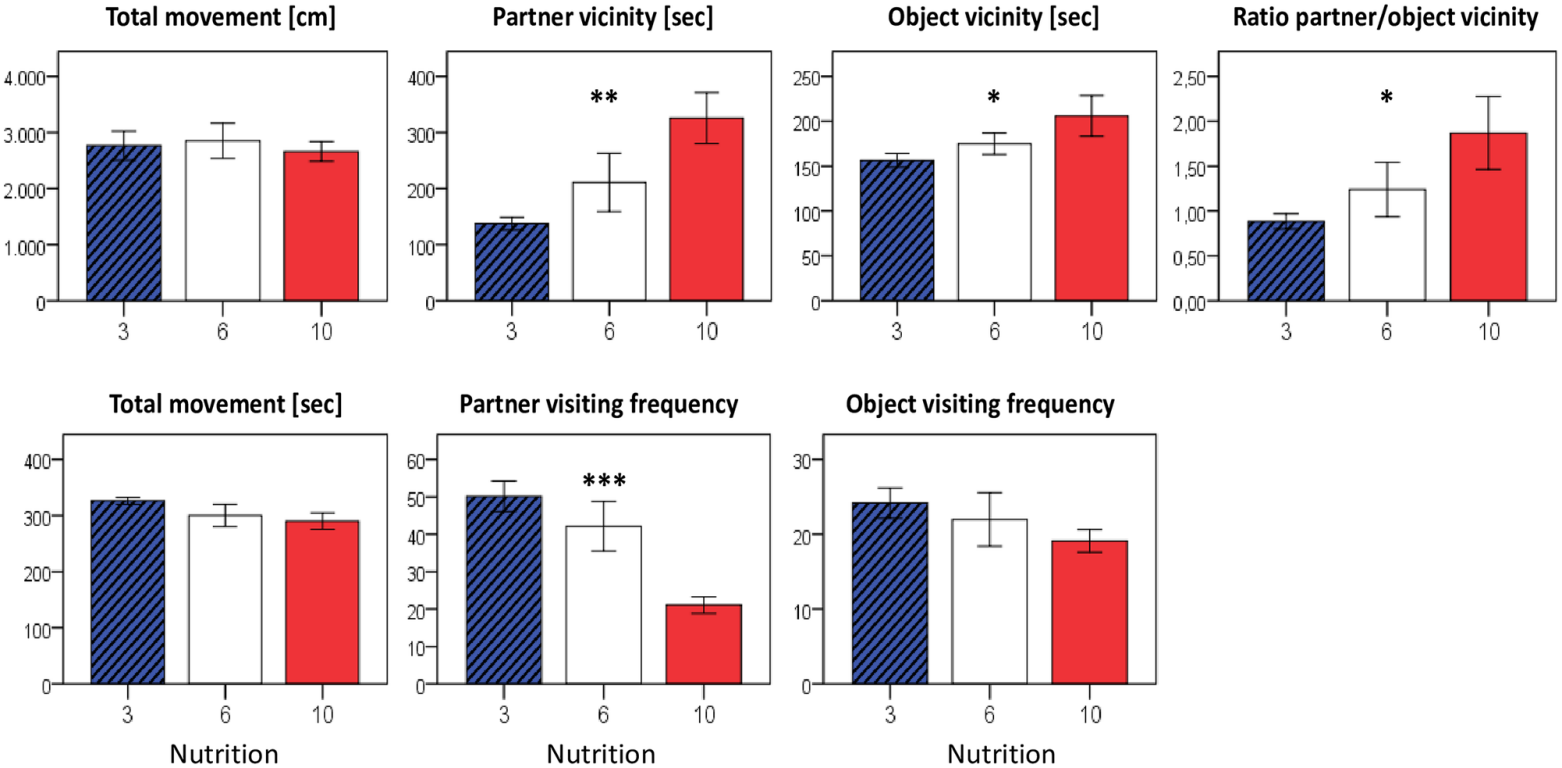
Open field experiment investigating social behavior at P45-P50. During a 10 min recording time, substantial differences were observed for parameters assessing social interaction with an unknown partner animal. Although formerly overfed males exhibited the highest partner visiting frequency, they spent the least time in the vicinity of the partner. Locomotor activity was not different between the nutrition groups. Data are presented as mean ±SEM (n = 6–8 males per nutrition group). If not indicated otherwise, displayed significance levels (* p<0.05, ** p<0.01, *** p<0.001) refer to comparisons between all three nutrition groups (ANOVA).

Behavior testing by an open-field-setting that focuses on social interaction revealed significant differences between the nutrition groups (Fig 9). Formerly underfed mice did not only exhibit the largest time spent with exploration (sum of time spent in the vicinity of either partner or object (10>6>3: p < 0.01, not shown)), they also turned out to be the most sociable animals, showing the longest duration of stay in the vicinity of a foreign animal (10>6>3: p = 0.003). This was also reflected by a significant increase in the ratio partner-to-object vicinity time (10>6>3: p = 0.036). Vice versa, formerly overfed animals showed the highest frequency of partner animal visits but, once there, were be less competent in initiating a social interaction. Horizontal locomotor activity, which was recorded simultaneously, was not different between the nutrition groups (Fig 9).

## Discussion

In this study, we show that modification of early postnatal nutrition by a cross-fostering strategy permanently alters somatic growth, behavior and parameters of the endocannabinoid metabolism, indicating their developmental plasticity. Whereas the effects observed on somatic growth and hepatic IGF-I expression are well in line with previous reports on pre- and early postnatal programming of the GH-IGF-I-axis in rodents and humans [8–10], our study gives, for the first time, evidence that variation of early postnatal nutrition within naturally occurring ranges may permanently alter parameters of the eCB system.

In adipose tissue, eCB-synthesizing enzymes NAPE-PLD and DAGLα were upregulated in formerly overfed animals, who remained taller and heavier compared to their normal- and underfed littermates. This is in accordance with the assumption of a generally higher activation of the eCB system in conditions of overnutrition and obesity, and increased DAGLα expression was also shown in subcutaneous tissue of obese human subjects [28]. On the other hand, eCB-degrading enzymes MGL (fat and liver) and FAAH (liver) also tended to be upregulated in formerly overfed animals. eCB concentrations in the liver and in white adipose tissue, although statistically largely comparable between nutrition groups, tended to be slightly increased in formerly overfed animals (and *vice versa)* (Figs 6 and 7). These findings may indicate that the eCB homeostasis comprising synthesis, degradation, and actual eCB tissue concentrations is still balanced. However, a number of studies in mice and men reported on a diminished FAAH expression found in adipose tissue of obese subjects [28–31], although other groups found unchanged or even increased FAAH expression associated with obesity [32, 33]. Conflicting results on either a positive [33, 34] or a negative correlation [29, 30] between BMI and expression levels of eCB-degrading enzymes in adipose tissue were reported by previous studies, and a converse regulation of these enzymes has even been reported between different adipose tissue depots [33], which further underscores the complex and tissue-specific regulation of eCB-metabolizing enzymes. Interestingly, FAAH expression in visceral adipose tissue was shown to be negatively regulated by TNFα *in vitro* [31], indicating that a reduced FAAH activity may occur particularly in conditions when inflammatory, or rather metaflammatory activity is already present in adipose tissue.

Regarding CB1R expression in adipose tissue and liver, we did not find a significant association with postnatal nutrition and subsequent somatic development. Several groups reported reduced levels of CB1R expression in adipose tissue of obese subjects, a finding that apparently contradicts with the conception of an overactivated eCB system in obesity and might be explained by a negative feed-back regulation [29, 31]. On the other hand, the activation of the eCB system observed in obesity-related non-alcoholic fatty liver disease and cirrhosis goes clearly along with increased levels of CB1R gene expression in the liver [35, 36], and studies in rodents revealed a direct transcriptional upregulation of the hepatic CB1R expression by endogenous cannabinoids [37, 38]. There is also evidence that overactivation of the eCB system and its action via hepatic CB1R is crucially involved in the pathogenesis of diet-induced metabolic changes such as insulin and leptin resistance, even suggesting that pharmacological targeting of liver-specific CB1R might be an effective strategy in the treatment of obesity-related metabolic disorders in the future [36]. Thus, the fact that in our study peripheral CB1R expression was not yet significantly altered and eCB concentrations in liver and adipose tissue were only marginally influenced by the postnatal nutrition scheme may indicate that the animals are still metabolically healthy. This is in accordance with hepatic interleukin (IL)-6 and tumor-necrosis-factor alpha (TNFα) gene expression patterns showing a gradual increase of IL-6 expression with highest levels in formely overfed mice, which however did not display a simultaneous increase in TNFα expression (S5 Fig). In a cohort of children and adolescents, Kitsios et al. reported gradually increasing IL-6 levels in comparison of normal, overweight, and obese individuals. TNFα levels however were only elevated in those patients who already suffered from non-alcoholic fatty liver disease (NAFLD), although they did not discriminate between adolescents with and without aspects of metabolic syndrome or prediabetes[39]. In adult populations, TNFα has been linked to parameters of metabolic deterioration, steatohepatitis and liver fibrosis [31,40,41].

Hence, one could speculate that transcriptional downregulation of endocannabinoid-degrading enzymes, possibly in response to later emerging metaflammation as shown for FAAH and TNFα [31], may represent a critical step in the development of hepatic alteration and metabolic disorder in obese subjects. This downregulation, in turn, would be able to further aggravate the eCB dysregulation in terms of a vicious cycle.

Thus, it will be interesting to generate further cross-fostering cohorts in order to longitudinally follow up the changes in the eCB system in relation to the metabolic development. This may also clarify whether the observed differences in eCB-metabolizing enzymes in our animals are only adaptive to nutritionally determined differences in somatic growth and BMI-development or may even turn out to become metabolic risk factors, particularly in conditions of developing insulin resistance and metaflammation. Kappeler et al., who investigated parameters of the glucose metabolism in a similar cross-fostering mouse model (3, 6, and 10 pups/mother until day 28), observed significantly higher glucose and insulin levels in 10 days-old overfed animals (3/mother) compared to controls (6/mother). Shortly thereafter, at age 20 days, glucose and insulin levels were almost equalized between these groups. However, formerly overfed animals exhibited significant hyperinsulinemia and reduced glucose tolerance in an *i*.*p*.GTT performed after 3 months, both of which further aggravated until the age of 1 year [10].

The observed permanent alteration of somatic growth after early postnatal relative over- and undernutrition is well in line with previous reports on nutritional programming of the GH-IGF-I-axis during early developmental periods in humans and rodents [8, 10]. However, little is known on a putative interaction between the GH-IGF-I-axis and the eCB system. Evidence for a direct interaction of endocannabinoids with the hepatic IGF-I expression comes from experiments with zebrafish. Anandamide (AEA) administration *via* water acutely upregulated the gene transcription of both IGF-I and IGF-II [42]. Furthermore, Al-Massadi and co-workers demonstrated that blockade of CB1R function inhibited the pulsatile GH secretion from the pituitary. The authors used different ways of rimonabant administration to investigate the mechanism by which CB1R blockade impairs GH secretion. Peripheral blockade via *i*.*p*. rimonabant led to a decrease in ghrelin-inducible GH secretion, with decreased levels of hypothalamic GHRH-expression. Interestingly, these effects were not reproducible by a central CB1R blockade, when rimonabant was administered *i*.*c*.*v*., and vagotomy was able to eliminate the inhibitory action of *i*.*p*. rimonabant on GHRH and GH. The authors conclude that endocannabinoids and peripheral CB1R in the vagal nerve play a crucial role for the eCB-related control of GH secretion [43].

Notably, we observed a significantly reduced CB1R gene expression in the arcuate nucleus of animals belonging to the nutrition-restricted group, as compared to formerly overfed animals (Fig 8). To our knowledge, no experimental data on hypothalamic endocannabinoid receptors and their local impact on GHRH-neurons are available to date. However, considering that in the study of Al-Massadi et al., GHRH expression in the arcuate nucleus as well as GHRH-induced GH-response at the pituitary level were affected only by peripheral CB1R blockade and shown to remain intact after *i*.*c*.*v*. rimonabant treatment, local CB1R in the arcuate nuclus may not have a major role in the hypothalamic regulation of the GH-IGF-I-axis [43]. Consequently, we cannot estimate whether the detected hypothalamic expression differences for CB1R are relevant for the somatotropic function.

However, other key functions of the arcuate nucleus may be affected by differences in eCB sensitivity, particularly appetite regulation. Certainly, a limitation of our study is that we did not quantify nutrient intake after weaning, redistribution and offering standard chow from P21 onwards. This would have been interesting since appetite regulation itself may be subject to learning processes or metabolic programming during early postnatal periods. However, in the aforementioned study of the group around L. Kappeler, who used a similar cross-fostering strategy in 129/ScPas—C57BL/6J hybrid mice, nutrient intake was quantified at the age of 3 months and did not significantly differ. Indeed, formerly overfed animals even had a slightly lower relative food intake compared to normal and underfed mice at this time [10].

Taken together, several parameters of the eCB system and the GH-IGF-I-axis were found to be permanently modified as a result of early postnatal over- or undernutrition in our study. There is increasing evidence for a functional crosstalk between these two complex biological systems, especially at peripheral levels. In terms of the concept of developmental programming of somatic growth, further studies are required to clarify whether and to which extent tissue-specific modifications of the eCB system may have a direct impact on the GH-IGF-I axis regulation.

Behavior testing in an open-field setting revealed significant differences between the nutrition groups, with formerly underfed mice turning out to be the most sociable animals. They spent significantly more time in the vicinity of a formerly unknown partner animal compared to control- and postnatally overfed mice. Based on previous reports [29] and own expression data for eCB-metabolizing enzymes, especially in adipose tissue, we assume that in our animals early postnatal overnutrition led to an increased eCB synthesis and the other way round. Assuming a stimulative effect of cannabinoid action on social activity [44, 45], a priori one could assume to find highest social interaction times in the overfed group. However, the opposite distribution was observed here and we can only speculate about the potential involvement of eCB effects. In our study, we have analyzed mRNA expression levels of eCB-metabolizing enzymes and receptors in only a small subset of metabolically active tissues, which may not correspond to the expression levels at those brain regions implicated in the modulation of social behavior. Moreover, measurement of eCB concentrations in the liver and in white adipose tissue, which may serve as a rough estimate of circulating eCB levels, revealed largely comparable eCB levels between nutrition groups, with a tendency towards increased eCB tissue concentrations in formerly overfed animals (and *vice versa*). Thus, the observed differences in social behavior traits may particularly result from a differential spatial modulation of eCB sensitivity via eCB receptors, i.e., a locally diminished CB1R expression in formerly overfed animals (as shown here for the arcuate nucleus) would indeed correspond to the aforementioned concept. Furthermore, we have to consider that postnatal litter size itself and different levels of maternal licking and grooming may have a direct impact on later social behavior [46]. However, Fairless et al. reported that C57BL/6J mice from larger litter sizes during the first four days of tended to be less sociable [47], a finding that conflicts with the observed distribution in our experiments. Social interactions tests are considered to be susceptible to context-effects such as anxiety, and the modulating effect of the eCB system on social interactions may be primarily context rather than behavior specific [45, 48]. In Sprague-Dawley rats, Dimitsantos and co-workers demonstrated that animals from smaller litters (n<10) showed higher scores of anxiety compared to animals from control (n = 10–15) or larger litter sizes (n>15) [49]. Finally, alterations of other neuroendocrine systems including neuroactive steroids and the hypothalamus-pituitary-adrenal gland (HPA)–axis [46, 50], which may be subject to developmental programming, too, could explain the observed differences in social behavior following the early postnatal cross-fostering period.

To conclude, we used a cross-fostering mouse model enabling us to show that early postnatal relative over- and undernutrition within naturally occurring ranges of maternal care is able to induce a permanent modification of somatic growth, behavior and parameters of the eCB system in metabolically active tissues. Further research is warranted in order to investigate whether developmental programming of the eCB system may be causally implicated in the formation of later metabolic health and disease, particularly after conditions of pre- or perinatal malnutrition which is frequently observed in infants born premature and/or small for gestational age.

## Supporting information

S1 Table Oligonucleotides used for real time PCR reactions. (PDF)

S1 Fig Melting curves of the real time PCR products corresponding to the oligonucleotides in S1 Table. Note that melting curves for primer pairs DAGLα and CB1R included small second peaks. However, agarose gel analyses of the products with ethidium bromide staining revealed specific single bands.
(PDF)

S2 Fig Representative real time PCR amplification curves for CB1R und CB2R from hypothalamic arcuate nucleus derived cDNA samples, showing low but detectable CB2R expression compared to CB1R. (PDF)

S3 Fig Social behavior testing using an open-field setting with two cage-in-cage boxes. One box contains a foreign ‘partner animal’, the other is empty and serves as control ‘object’. Movements of the animal are recorded during a 10 min period (6–8 males per nutrition group).
(PDF)

S4 Fig Auxological parameters at P21, P50 and P100 (data from two separate animal cohorts, ‘P50’ and ‘P100’). (PDF)

S5 Fig Relative gene expression of interleukin (IL)-6 and tumor-necrosis-factor alpha (TNFα) in the liver at P50. (PDF)

S1 Dataset Data underlying this study. (ZIP)
