# Supplementary material for: Developmental programming of somatic growth, behavior and endocannabinoid metabolism by variation of early postnatal nutrition in a cross-fostering mouse model
Source: PLoS One. 2017 Aug 31;12(8):e0182754. doi: 10.1371/journal.pone.0182754 (PMC5578498; doi:10.1371/journal.pone.0182754)
Supplement: S1 Table — (PDF) [file pone.0182754.s001.pdf]

Supporting information, Table S1
Oligonucleotides used for Real-Time PCR reactions

Primer	5‘3‘ nucleotide sequence
IGF-I P1 for	GCGATGGGGAAAATCAGCAG
IGF-I P1 rev	GAAGAGCATCCACCAGCTCA
IGF-I P2 for	AGTACCCACTCTGACCTGCT
IGF-I P2 rev	GAAGAGCATCCACCAGCTCA
CB1R for	TTCCTTGTAGCAGAGAGCCAG
CB1R rev	CTCAACGTGACTGAGAAAGAGG
CB2R for	TGACAAATGACACCCAGTCTTCT
CB2R rev	ACTGCTCAGGATCATGTACTCCTT
NAPE-PLD for	CAGGTTCCAAAGAGGAACTTGAC
NAPE-PLD rev	GATGAGCTCGTCCATTTCCAC
DAGLα for	GATTCCCAGTCAGATGCCTAC
DAGLα rev	GACAGGAAAGCCAAGATGTC
FAAH for	GAGATGTATCGCCAGTCCGT
FAAH rev	ACAGGCAGGCCTATACCCTT
MGL for	TCTTCCTCCTGGGCCACT
MGL rev	AAAGTAGGTTGGCCTCTCTGC
IL-6 for	ACGGCCTTCCCTACTTCACA
IL-6 rev	CATTTCCACGATTTCCCAGA
TNFα for	GCCTCTTCTCATTCCTGCTTG
TNFα rev	CTGATGAGAGGGAGGCCATT
βActin for	GTGATGGTGGGAATGGGTCA
βActin rev	CTGGATGGCTACGTACATGG
