# Supplementary material for: Developmental programming of somatic growth, behavior and endocannabinoid metabolism by variation of early postnatal nutrition in a cross-fostering mouse model
Source: PLoS One. 2017 Aug 31;12(8):e0182754. doi: 10.1371/journal.pone.0182754 (PMC5578498; doi:10.1371/journal.pone.0182754)

Supporting information, Fig. S1

Melting curves of the real time PCR products

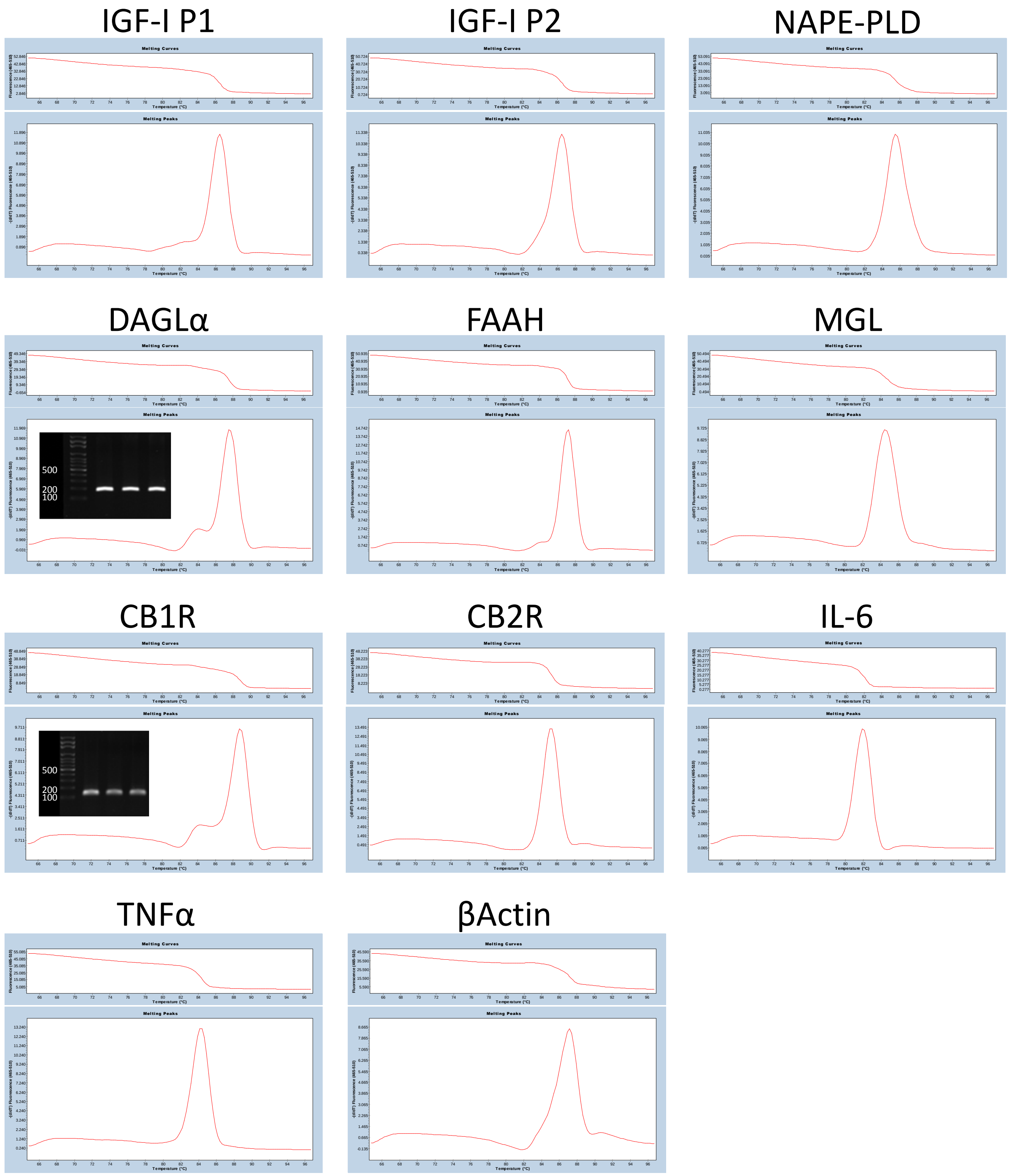

Supplement: S1 Fig — Note that melting curves for primer pairs DAGLα and CB1R included small second peaks. However, agarose gel analyses of the products with ethidium bromide staining revealed specific single bands. (PDF) [file pone.0182754.s002.pdf]
