# Supplementary material for: Developmental programming of somatic growth, behavior and endocannabinoid metabolism by variation of early postnatal nutrition in a cross-fostering mouse model
Source: PLoS One. 2017 Aug 31;12(8):e0182754. doi: 10.1371/journal.pone.0182754 (PMC5578498; doi:10.1371/journal.pone.0182754)

Supporting information, Fig. S2

Representative real time PCR amplification curves from hypothalamic arcuate nucleus RNA derived cDNA samples, showing low CB2R expression compared to CB1R.

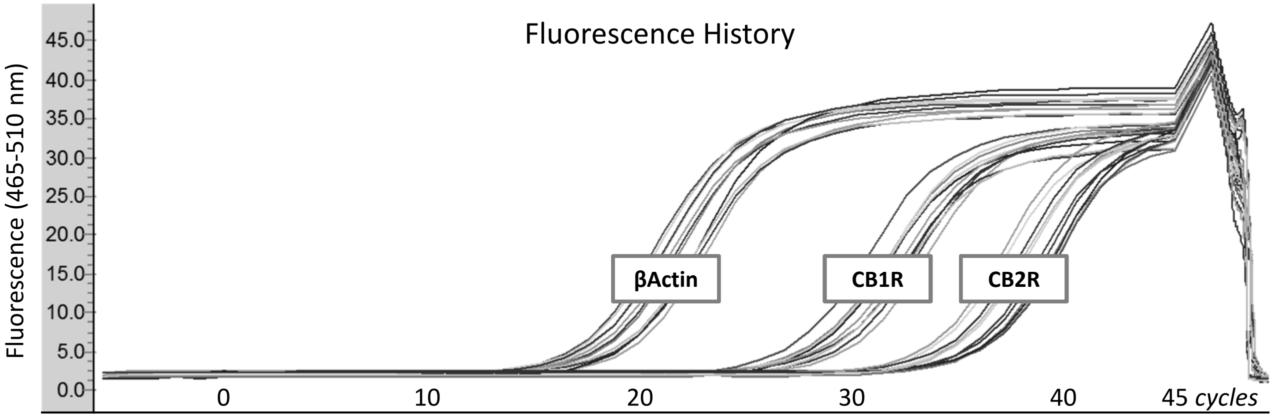

Supplement: S2 Fig — (PDF) [file pone.0182754.s003.pdf]
