# Supplementary figures and images for: Developmental programming of somatic growth, behavior and endocannabinoid metabolism by variation of early postnatal nutrition in a cross-fostering mouse model
Source: PLoS One. 2017 Aug 31;12(8):e0182754. doi: 10.1371/journal.pone.0182754 (PMC5578498; doi:10.1371/journal.pone.0182754)

Supporting information, Fig. S3

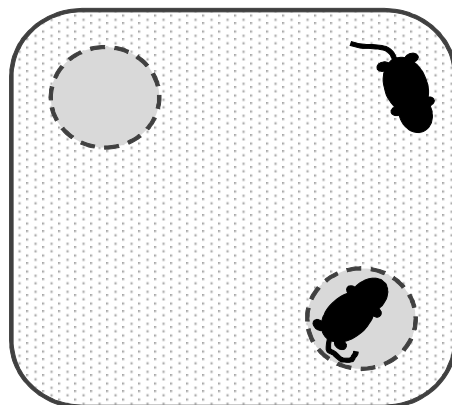

Supplement: S3 Fig — One box contains a foreign ‘partner animal’, the other is empty and serves as control ‘object’. Movements of the animal are recorded during a 10 min period (6–8 males per nutrition group). (PDF) [file pone.0182754.s004.pdf]
