# Supplementary material for: Developmental programming of somatic growth, behavior and endocannabinoid metabolism by variation of early postnatal nutrition in a cross-fostering mouse model
Source: PLoS One. 2017 Aug 31;12(8):e0182754. doi: 10.1371/journal.pone.0182754 (PMC5578498; doi:10.1371/journal.pone.0182754)

Supporting information, Fig. S4

Auxological parameters at P21, P50 and P100 (data from two separate animal cohorts, 'P50' and 'P100')

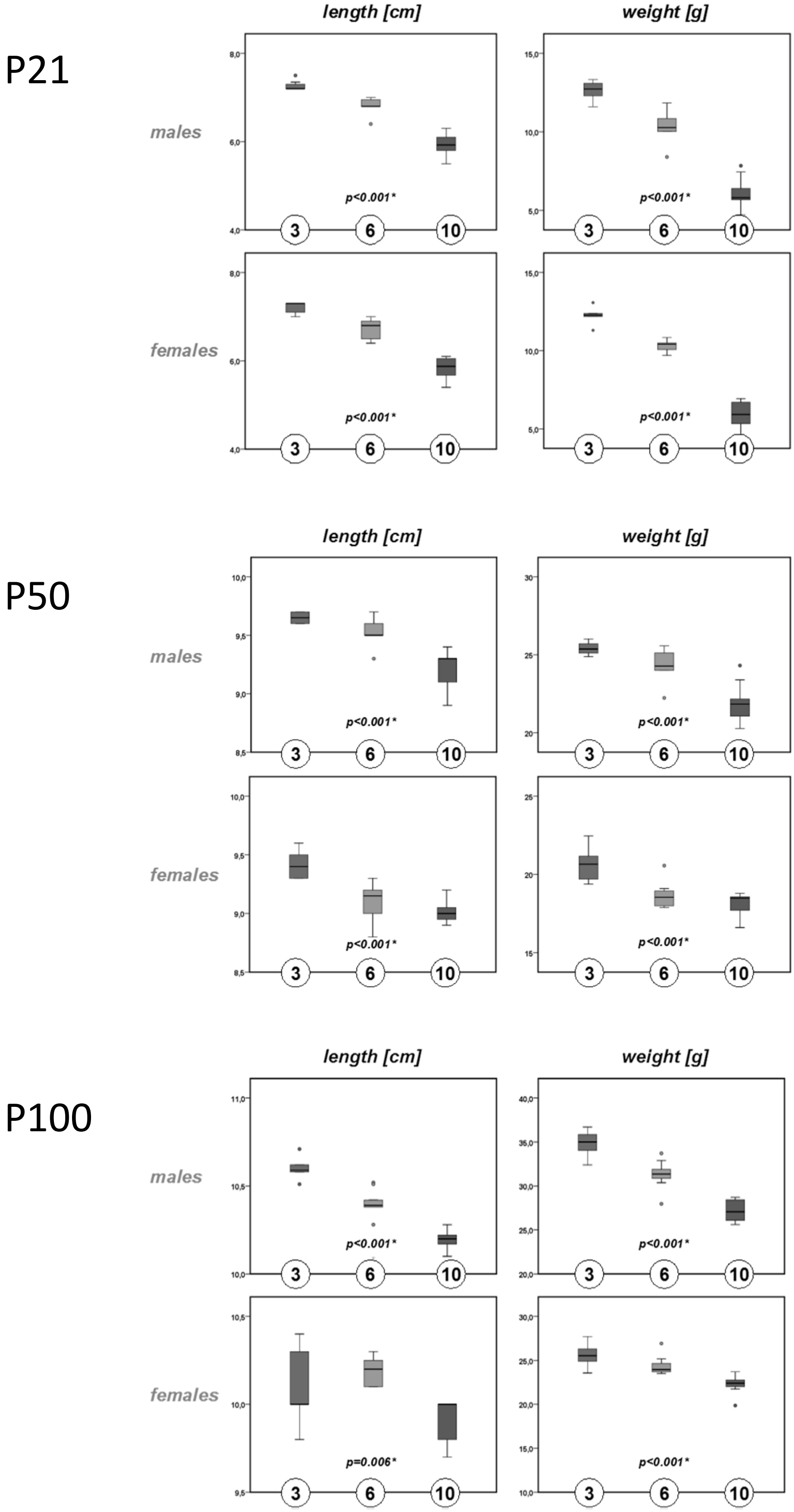

Supplement: S4 Fig — (PDF) [file pone.0182754.s005.pdf]
