# Supplementary material for: Developmental programming of somatic growth, behavior and endocannabinoid metabolism by variation of early postnatal nutrition in a cross-fostering mouse model
Source: PLoS One. 2017 Aug 31;12(8):e0182754. doi: 10.1371/journal.pone.0182754 (PMC5578498; doi:10.1371/journal.pone.0182754)

Supporting information, Fig. S5

Relative gene expression of interleukin (IL)-6 and tumor-necrosis-factor alpha (TNFα) in the liver at P50 in relation to early postnatal nutrition. n= 4-6 males per nutrition group, ** p<0.01, *** p<0.001 (ANOVA).

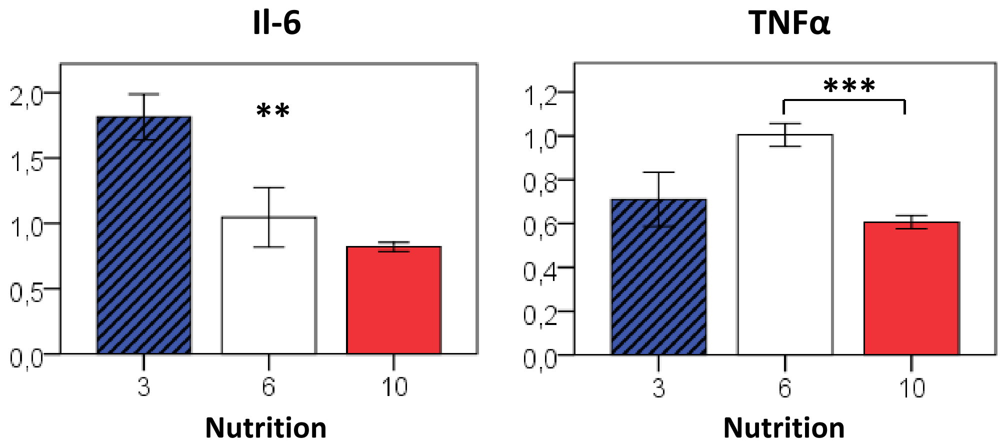

Supplement: S5 Fig — (PDF) [file pone.0182754.s006.pdf]
